# Quantification of epicardial fat using 3D cine Dixon MRI

**DOI:** 10.1186/s12880-020-00478-z

**Published:** 2020-07-14

**Authors:** Markus Henningsson, Martin Brundin, Tobias Scheffel, Carl Edin, Federica Viola, Carl-Johan Carlhäll

**Affiliations:** 1 Division of Cardiovascular Medicine, Department of Medical and Health Sciences, Linköping University, Linköping, Sweden; 2 Center for Medical Image Science and Visualization (CMIV), Linköping University, Linköping, Sweden; 3 School of Biomedical Engineering and Imaging Sciences, King’s College London, London, UK; 4 Department of Clinical Physiology, Department of Medical and Health Sciences, Linköping University, Linköping, Sweden

**Keywords:** Dixon, Epicardial fat, Cine MRI, Whole-heart imaging

## Abstract

**Background:**

There is an increased interest in quantifying and characterizing epicardial fat which has been linked to various cardiovascular diseases such as coronary artery disease and atrial fibrillation. Recently, three-dimensional single-phase Dixon techniques have been used to depict the heart and to quantify the surrounding fat. The purpose of this study was to investigate the merits of a new high-resolution cine 3D Dixon technique for quantification of epicardial adipose tissue and compare it to single-phase 3D Dixon in patients with cardiovascular disease.

**Methods:**

Fifteen patients referred for clinical CMR examination of known or suspected heart disease were scanned on a 1.5 T scanner using single-phase Dixon and cine Dixon. Epicardial fat was segmented by three readers and intra- and inter-observer variability was calculated per slice. Cine Dixon segmentation was performed in the same cardiac phase as single-phase Dixon. Subjective image quality assessment of water and fat images were performed by three readers using a 4-point Likert scale (1 = severe; 2 = significant; 3 = mild; 4 = no blurring of cardiac structures).

**Results:**

Intra-observer variability was excellent for cine Dixon images (ICC = 0.96), and higher than single-phase Dixon (ICC = 0.92). Inter-observer variability was good for cine Dixon (ICC = 0.76) and moderate for single-phase Dixon (ICC = 0.63). The intra-observer measurement error (mean ± standard deviation) per slice for cine was − 0.02 ± 0.51 ml (− 0.08 ± 0.4%), and for single-phase 0.39 ± 0.72 ml (0.18 ± 0.41%). Inter-observer measurement error for cine was 0.46 ± 0.98 ml (0.11 ± 0.46%) and for single-phase 0.42 ± 1.53 ml (0.17 ± 0.47%). Visual scoring of the water image yielded median of 2 (interquartile range = [Q3-Q1] 2–2) for cine and median of 3 (interquartile range = 3–2) for single-phase (*P* < 0.05) while no significant difference was found for the fat images, both techniques yielding a median of 3 and interquartile range of 3–2.

**Conclusion:**

Cine Dixon can be used to quantify epicardial fat with lower intra- and inter-observer variability compared to standard single-phase Dixon. The time-resolved information provided by the cine acquisition appears to support the delineation of the epicardial adipose tissue depot.

## Background

The volume of epicardial adipose tissue has been linked to increased risk of cardiovascular disease such as atrial fibrillation [1–3], atherosclerosis [4, 5], ventricular dysfunction [6–8], and improves prediction of coronary artery disease [9]. As a result, there is a growing interest in depicting and quantifying the amount of epicardial adipose tissue [10, 11]. In recent years, cardiovascular magnetic resonance techniques relying on Dixon water-fat separation have been proposed to this end [12–14]. The Dixon technique allows quantification of both epicardial and paracardial fat, which are separated by the parietal pericardium [15]. While the epicardial fat produce adipokines, which can diffuse into the adjacent myocardium and coronary arteries and directly trigger a pathophysiologic response, the role of the more remote paracardial fat is less well-understood [16]. A previous study has shown the volume of epicardial and paracardial fat to be highly correlated, suggesting a combined cardiac fat measurement may be sufficient to infer either cardiac fat component [14]. However, the outer boundaries of the paracardial fat may be challenging to define, potentially leading to higher user dependence, and may require an increased field-of-view and hence scan time to capture compared to standard whole-heart protocols. Therefore, it is desirable to achieve a robust method for depicting and quantifying the epicardial fat, which is likely the cardiac fat component with most predictive power for cardiovascular disease, with low operator dependence. The currently used method for cardiac fat quantification using cardiovascular magnetic resonance (CMR) relies on a high-resolution 3D multi-echo Dixon technique [14, 17, 18]. Data acquisition is limited to a motion-free cardiac phase to minimize cardiac motion artifacts, while navigator gating is used to reduce respiratory motion artifacts. Despite relatively high spatial resolution (approximately 1.5 mm in-plane) and motion compensation, adequate spatial separation between the epicardial fat and paracardial fat boundaries may be difficult to achieve with this approach. However, to aid the differentiation of epicardial and paracardial fat depots the differences in cardiac motion between these two categories may be exploited. The epicardial fat, which is attached to the moving heart, is closely related to the cardiac motion, while the paracardial fat has a relatively smaller deformation due to cardiac motion [19]. This difference may be captured using time-resolved cine CMR where data acquisition is performed throughout the cardiac cycle and multiple cardiac phases retrospectively reconstructed. We hypothesize that the time-resolved information provided by the new cine Dixon technique facilitates visualization of epicardial fat and leads to better quantification reproducibility compared to standard single-phase Dixon.

Accordingly, in this proof-of-concept study we investigate the merits of a new high-resolution cine 3D Dixon technique for quantification of epicardial adipose tissue and compare it to the standard single-phase 3D Dixon in patients with cardiovascular disease.

## Methods

### Study population

Fifteen consecutive patients referred to our hospital for clinical CMR examination of known or suspected heart disease were considered for inclusion in the study. The median patient age was 55 years and ranged from 24 to 72 years. The demographics and basal clinical characteristics of all patients are described in Table 1. All patients provided written informed consent prior to participation and the study was approved by the regional ethics committee. Patients were included in the study if the CMR protocol required the use of contrast agent, as the 3D cine Dixon water image benefits greatly from contrast enhancement due to increased signal-to-noise ratio of the blood pool. No contraindications were imposed specifically for the 3D cine Dixon scan.

**Table 1:** Patient characteristics

Patient	Gender (M/F)	BMI (kg/m^2^)	HR (bpm)	LVEF (%)	LVEDV (ml)	Diagnosis/symptoms
P1	F	23.3	81	66	118	Dyspnea, palpitations
P2	M	24.9	96	65	179	Atrial fibrosis with arrhythmia
P3	M	29.0	82	55	100	Myocarditis
P4	M	28.1	59	40	437	Severe aortic regurgitation
P5	M	27.1	65	57	196	Moderate aortic regurgitation
P6	F	26.6	58	68	160	Non-compaction cardiomyopathy
P7	M	24.2	59	66	187	Stroke
P8	M	22.1	58	57	128	Arrhythmia
P9	F	26.0	58	60	166	Myocardial infarction
P10	M	27.4	71	58	163	Non-compaction cardiomyopathy
P11	M	22.1	58	28	305	DCM, myocardial infarction
P12	F	25.8	90	67	119	Chest pain, dyspnea
P13	M	39.6	103	34	250	Heart transplantation, CAD
P14	M	24.5	68	38	178	Myocardial infarction
P15	F	23.0	82	71	96	CAD

BMI, body max index; CAD, coronary artery disease; DCM, dilated cardiomyopathy; EDV, end-diastolic volume; EF, ejection fraction; HR, heart rate; LV, left ventricle;

### Data acquisition

All MRI examinations were performed on a 1.5 T Philips scanner (Philips Healthcare, Best, The Netherlands) using a 24-channel cardiac coil. The proposed 3D cine Dixon technique consisted of a 2-point spoiled gradient echo acquisition with an axial orientation. Imaging parameters included: field-of-view = 340 × 340 × 130–150 mm; acquired voxel size = 1.5 × 1.5 × 3.0 mm; reconstructed voxel size = 0.8 × 0.8 × 1.5 mm; flip angle = 10°; TR = 5.0 ms; TE_1_/TE_2_ 2.3/3.9 ms; bandwidth = 936 Hz/pixel. The temporal resolution of the cine Dixon scan was 65 ms, resulting in approximately 15 frames per cycle for a heart rate of 60 bpm. A respiratory navigator was used for respiratory motion compensation. The navigator was performed at the beginning of each cardiac cycle, immediately after the detection of the R-wave, and the navigator signal was used to gate the scan to end-expiration with an acceptance window of 5 mm. A commercially available compressed sensing technique was employed for image acceleration using a wavelet transform to enforce sparsity with a total acceleration factor of 5. With these imaging parameters, the nominal scan time was 2 min and 45 s, assuming a heart rate of 60 bpm. The 3D cine Dixon scan was performed immediately following contrast agent injection (0.2 mmol/kg gadobutrol) to improve contrast in the water image.

For comparison, a single-phase 3D Dixon scan was performed with identical field-of-view, voxel size, TR, TE_1_, TE_2_, and acceleration factors. The main differences included a larger flip angle of 20°, T2prep with an echo time of 20 ms to suppress signal from the myocardium, and data acquisition restricted to the mid-diastolic rest period of approximately 80–120 ms, as identified from a four-chamber 2D cine scan. The T2 prep was used to improve contrast between the blood-pool and the myocardium in the water images. With these imaging parameters the single-phase Dixon scan time was 1 min and 50 s. The 3D single-phase Dixon scan was performed immediately after the 3D cine Dixon.

### Image analysis

**Fig. 1:**
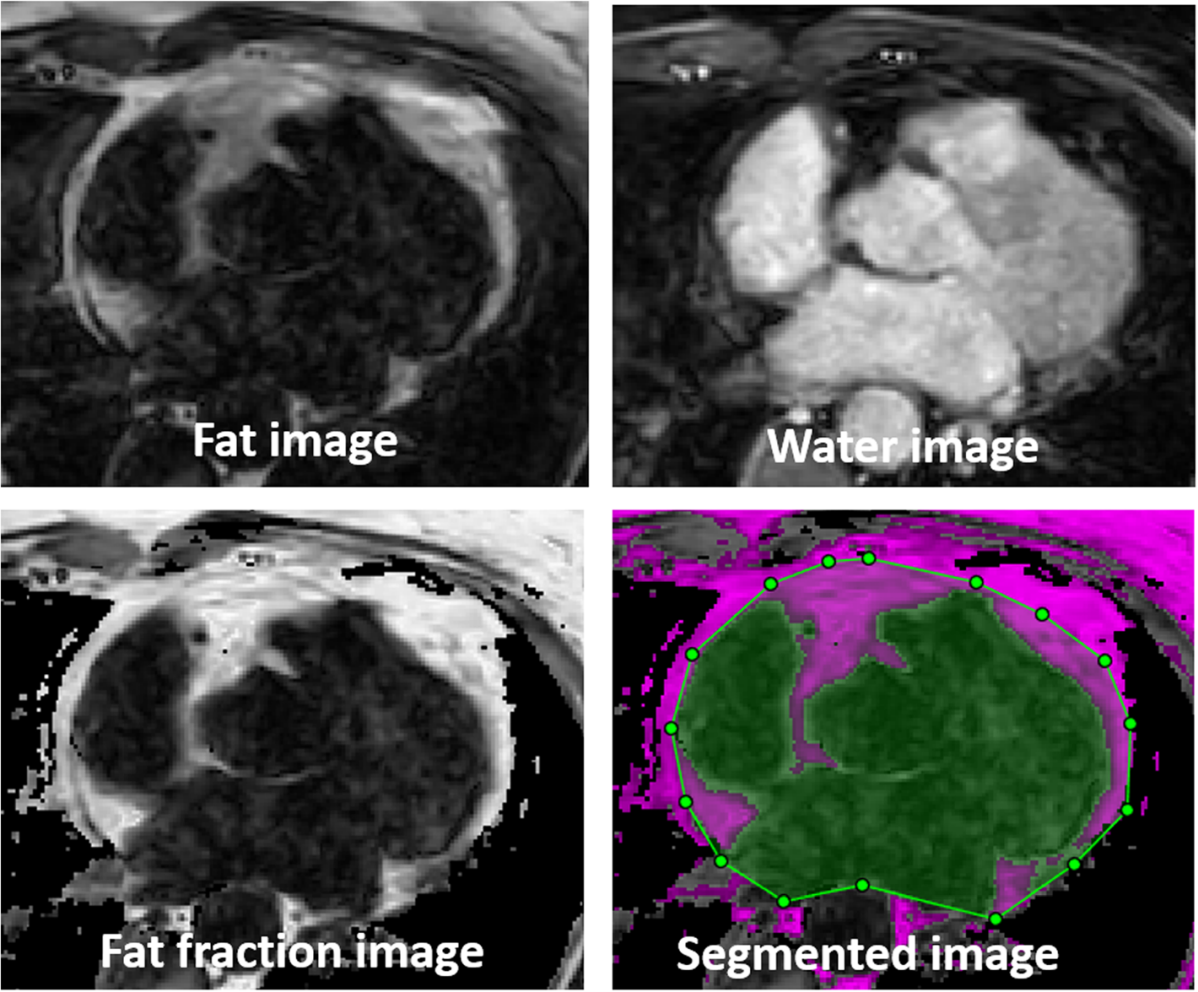
Reconstructed fat, water, fat fraction and segmented images from a cine Dixon scan. The fat fraction was calculated as the proportion of fat signal relative to water signal for each voxel. The manual segmentation indicated by the purple pixels within the green region-of-interest was performed using the fat fraction image and involved tracing the outer border of the epicardial fat. All cine timeframes were used to support the segmentation process. However, segmentation was only performed in a single timeframe, corresponding to the single phase time frame

The images were transferred to a separate workstation for analysis using customized software implemented in Matlab (Mathworks, Natick, MA). The outer epicardial boundaries were manually outlined for each slice using the reconstructed water (W) and fat (F) image intensities, and a fat-fraction (FF) image which was reconstructed using the formula FF=F/(F + W) for each voxel [14]. The FF image was used to facilitate fat volume classification where FF above a certain threshold was classified as fat. The reconstructed water, fat and fat-fraction images and segmentation process for one patient is shown in Fig. 1. Classification of pixels as epicardial fat was performed using the FF image where only pixels within the manually defined epicardial border were considered. A threshold operation was applied to remove pixels within the cardiac chambers and great vessels which have a low fat fraction. This segmentation procedure was performed for all slices in the axial stack between the last slice showing myocardial (apical) tissue in caudal direction and the first slice showing the pulmonary bifurcation in the cranial direction. The total epicardial fat volume for each patient was obtained by integrating the segmented epicardial fat across all slices. For the cine Dixon the segmentation was only performed in a single timeframe, corresponding to the same cardiac phase as the single-phase Dixon. The trigger delay of the single-phase scan was recorded, and the corresponding cine frame was determined by finding its closes match. For the cine Dixon segmentation all cine timeframes were available for visual inspection to support the delineation of the epicardial border in the chosen time-frame. The same fat-fraction threshold was used for the single-phase and cine Dixon, which was empirically determined to 0.35 (fat fraction pixels over 35% were classified as fat).

The fat quantification, as outlined above, was performed by three observers (TS, MB and CE), each segmenting single-phase and cine Dixon data in random order from five patients. TS (1 year of CMR experience), MB (2 years CMR experience) and CE (1 year of CMR experience) performed all quantitative analyses after receiving 10–15 h of one-on-one training and feedback by CJC (with 20 years of experience in CMR). To allow inter-observer variability analysis, the segmentation of five patients performed by TS was also performed by MB. The five patients were randomly selected from the 15 and their clinical indication were: myocarditis, non-compaction cardiomyopathy, 2 × coronary artery disease, and one patient with chest pain and dyspnea. For intra-observer variability, TS segmented data from the same five patients two times with a period of 7 days in between. For the inter- and intra-observer variability analysis out of the approximately 100 available slices (150 mm field-of-view in slice-direction with 1.5 mm resolution) for each patient and dataset, every 10th slice was used in the analysis, which is sufficiently spaced to be considered separate independent segmentations. In total, this yielded 50 data points for each method (single-phase and cine) for the inter- and intra-observer analysis.

To compare image quality between the proposed cine Dixon technique and the conventional single-phase approach, the water and fat images were separately visually scored by three experienced readers (CJC, MH with 12 years of CMR experience and CE) blinded to the acquisition method used. A consensus score was agreed upon by the three readers for each image. Similar to the fat segmentation described previously, only the timeframe from the cine Dixon acquisition corresponding to the single-phase was used for this comparison. The visual scoring was performed using a 4-point Likert scale which was defined as follows: 1 = severe blurring of cardiac structures; 2 = significant blurring of cardiac structures; 3 = mild blurring of cardiac structures; and 4 = no blurring of cardiac structures.

### Statistical analysis

All statistical analysis was performed using Matlab (Mathworks, Natick, MA). A Bland-Altman analysis was performed to assess inter- and intra-observer variability. Measurement errors per slice was calculated as the mean difference and standard deviations between observations, expressed in both actual measurement units (ml) and percentages. Intra-class correlation coefficient (ICC) was also calculated to determine agreement between measurements. Coefficients below 0.5 were considered poor, between 0.5 and 0.75 moderate, between 0.75 and 0.9 good, and above 0.9 excellent [20]. Linear regression analysis was performed for the relationship between body mass index (BMI) and the measured epicardial fat volumes normalized by the body surface area (BSA) using either single-phase or cine Dixon. Continuous variables were compared using a t-test while non-parametric variables were compared using Wilcoxon signed-rank test. All values are given as group mean ± 1 SD unless otherwise specified. A threshold of *P* < 0.05 was used to define statistically significant differences.

## Results

**Fig. 2:**
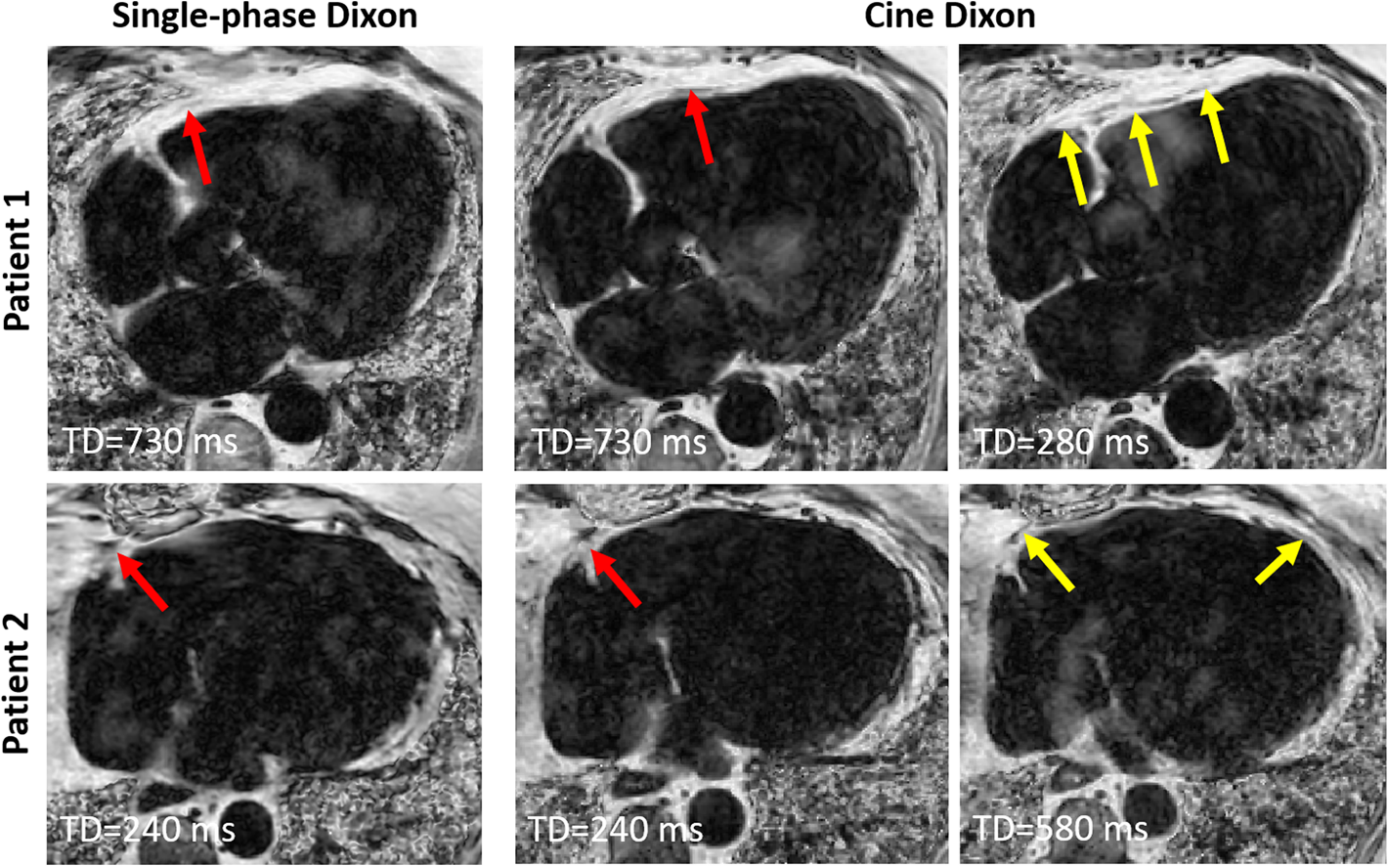
Fat fraction images from two patients where the parietal pericardium, separating the paracardial and epicardial fat depots can be only partially seen in the single-phase Dixon scans acquired during the diastolic rest period (trigger delay [TD] = 730 ms) of Patient 1 and diastolic rest period (TD = 240 ms) of Patient 2 (red arrows). The cine Dixon images depict a larger portion of the epicardial fat border in the complementary systolic/diastolic frames (yellow arrows)

**Fig. 3:**
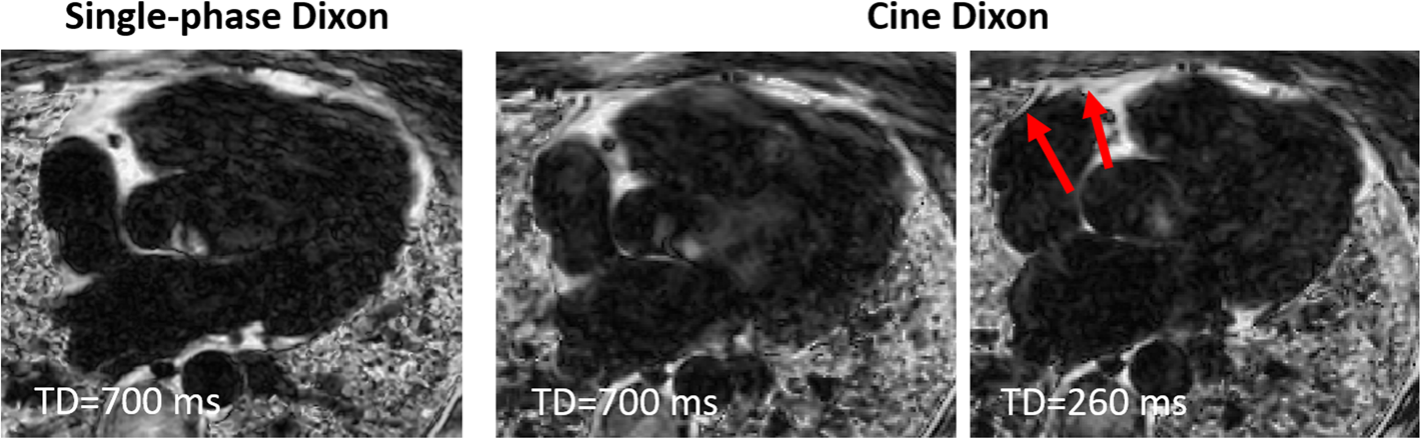
Fat fraction image of patient where the epicardial fat border along the right atrium and AV-groove is indistinguishable in both single-phase Dixon in diastole (TD = 700 ms) and cine Dixon in corresponding time frame in diastole (TD = 700 ms). However, in the cine Dixon, the epicardial fat border along the right atrium and AV-groove can be separated from the adjacent paracardial fat due to the AV-plane displacement in the apical direction during systole (arrows)

The single phase and cine Dixon scans were successfully performed in all 15 patients. The amount of fat measured using the cine Dixon scan was 145 ± 90 ml, and for the single-phase Dixon 165 ± 88 ml (*P* < 0.01). Example fat fraction images acquired using single-phase and cine Dixon are shown in Fig. 2, where cine Dixon allows for better delineation of the epicardial fat border in a systolic or diastolic frame which was not captured in the corresponding single-phase Dixon. Video files of the time-resolved fat-fraction images from the patients in Fig. 2 are provided in Additional file 1: Video S1 and Additional file 2: Video S2. An example of where the motion of the epicardial fat during the cardiac cycle, derived from the cine Dixon, was useful to separate epicardial from paracardial fat is shown in Fig. 3. A video file of the time-resolved fat-fraction image for the patient in Fig. 3 are provided in Additional file 3: Video S3 to further demonstrate the value of cine Dixon.

**Fig. 4:**
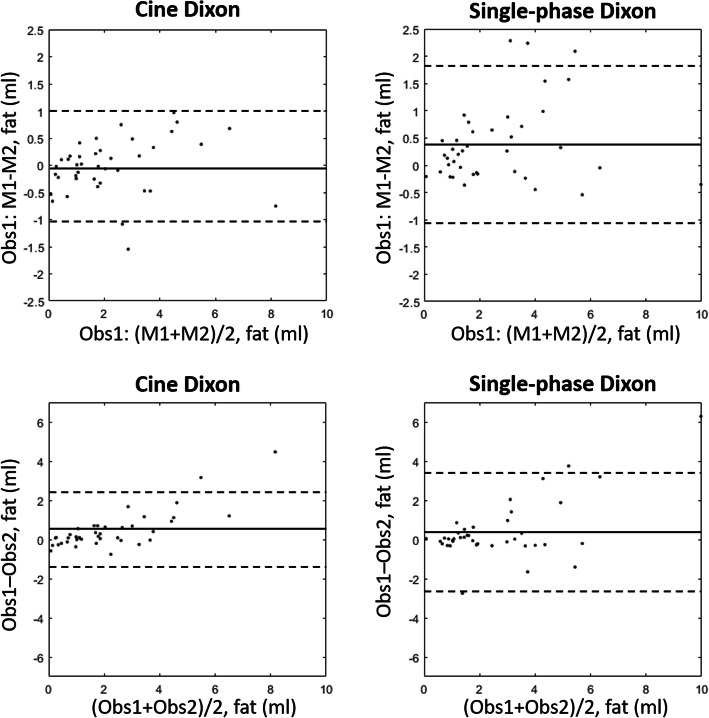
Bland-Altman plots of the intraobserver difference (top row) and interobserver difference (bottom row) for cine Dixon (left column) and single-phase Dixon (right) column. Mean (solid line) ± two standard deviations (dotted lines) are shown. Obs = observer; M = measurement

Bland-Altman plots for the inter- and intra-observer measurements using cine and single-phase Dixon are shown in Fig. 4. The intra-observer measurement error (as mean ± standard deviation) per slice for the cine Dixon scan was − 0.02 ± 0.51 ml (− 0.08 ± 0.4%), and for the single-phase Dixon 0.39 ± 0.72 ml (0.18 ± 0.41%). The inter-observer measurement error per slice for cine Dixon was 0.46 ± 0.98 ml (0.11 ± 0.46%) and for single-phase 0.42 ± 1.53 ml (0.17 ± 0.47%). Intra-observer variability using ICC was excellent for the cine images (ICC = 0.96), and slightly higher than for single-phase Dixon (ICC = 0.92). Inter-observer variability was good for cine Dixon (ICC = 0.76) and moderate for single-phase Dixon (ICC = 0.63).

The visual scoring of image quality for the water image yielded a median score of 2 (interquartile range [IQR: Q3-Q1] = 2–2) for cine Dixon and a median of 3 (IQR = 3–2) for single-phase Dixon. The improved image quality in favor of single-phase Dixon was statistically significant (*p* < 0.05). The image quality scoring of the fat images yielded a median of 3 (IQR = 3–2) for the cine Dixon images and a median of 3 (IQR = 3–2) for the single-phase Dixon (*p* = 0.32).

**Fig. 5:**
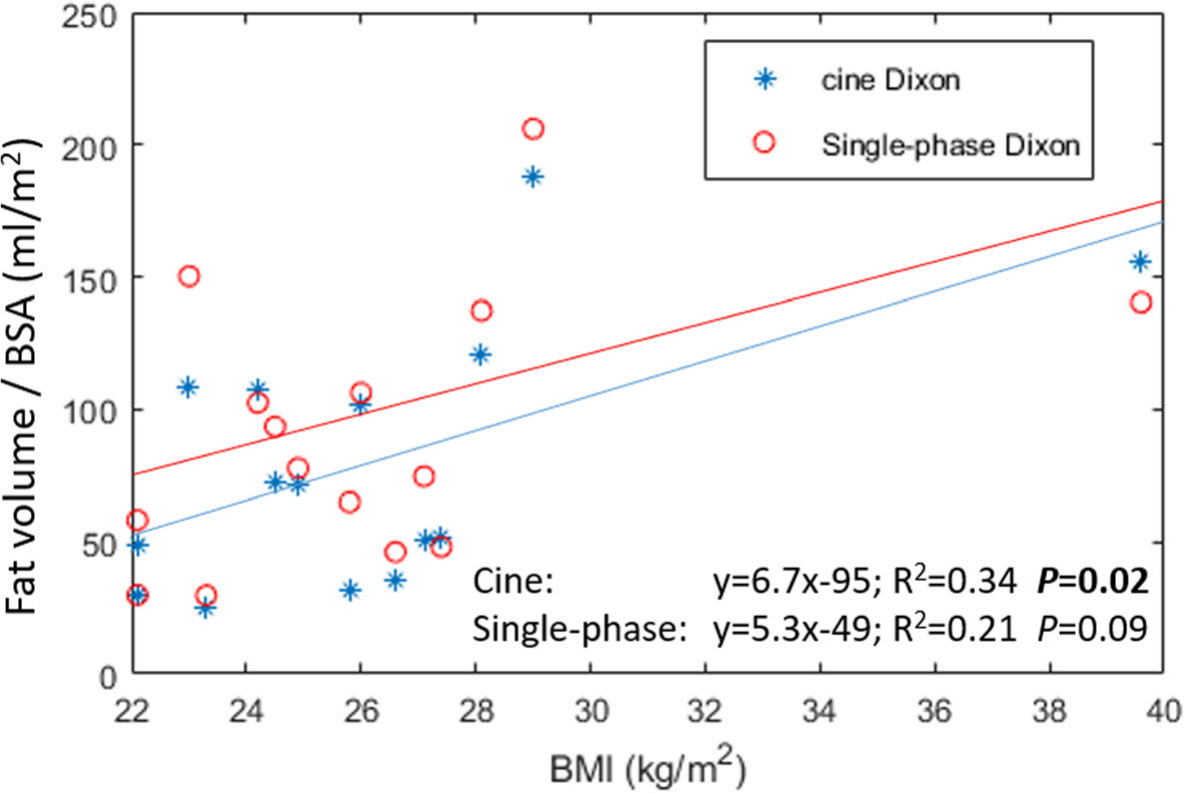
Linear regression analysis showing the relationship between body max index (BMI) and epicardial fat volume adjusted by body surface area (BSA)

The linear regression analysis for the correlation between BMI and fat volume indexed to body surface using cine Dixon and single-phase Dixon is shown in Fig. 5. The fat volumes obtained with the new cine Dixon technique showed a non-random but weak correlation (R^2^ = 0.34, *p* = 0.02) with BMI whereas the single-phase Dixon did not show any significant correlation with BMI (R^2^ = 0.21, *p* = 0.09).

## Discussion

In this study, we have implemented and evaluated a new 3D cine Dixon technique for the quantification of epicardial adipose tissue. Compared to standard single-phase Dixon, the proposed method yields fat quantification values with lower inter- and intraobserver variability.

The availability of images throughout the cardiac cycle allows retrospective selection of a phase with potentially better visualization of the epicardial fat border. Conversely, for the standard single-phase approach, the trigger delay is prospectively determined and the timing for optimal epicardial fat border detection is unknown. Examples of this have been provided (Fig. 2) where the single-phase Dixon acquired in the phase with longest rest period yield images with suboptimal delineation of the epicardial fat depot, while better visualization is obtained with cine Dixon in the opposite (systolic/diastolic) cardiac rest period. A corollary to this is that no specific timing information is required prior to the cine Dixon scan thus simplifying scan setup, while additional time-resolved 2D scans are needed to prospectively define the single-phase Dixon timing parameters [21].

The ability to visualize the motion of the epicardial fat may also provide information to help separate epicardial from paracardial fat, even if the border between the tissues is inconspicuous. This may be particularly beneficial for identifying the border of the epicardial fat near the atrioventricular groove and the basal part of the right ventricle, which typically experience significant longitudinal motion during the cardiac cycle relative to the more static adjacent paracardial fat. An example of this motion-related differentiation of epicardial and paracardial fat have been provided (Fig. 3 and supplementary video), and this advantage of cine Dixon may further help to explain why segmentation is less variable using this approach compared to single-phase Dixon.

In this study, we found a lower intra-and inter-observer variability using a novel cine Dixon technique despite slightly reduced image quality of the water image compared to conventional single-phase Dixon. Although there was no significant difference for the fat images, segmentation was performed using the fat fraction images which combines the water and fat images. However, the small reduction in image quality using cine Dixon is likely primarily due to residual motion artifacts. A diaphragmatic navigator was used to gate the cine acquisition to end-expiration and performed once per cardiac cycle, resulting in low temporal proximity to the image acquisition in many frames. However, both navigator gating and slice tracking was employed for single-phase Dixon, where the navigator was performed immediately prior to the data readout for each cardiac cycle. Recent improvements in respiratory motion compensation techniques for whole-heart cine such as self-navigation are likely to prove beneficial in combination with cine Dixon for epicardial border delineation and can be included in future work to further improve this technique [22–29].

The assessed epicardial fat volume was systematically higher using the single-phase Dixon sequence compared to the proposed cine Dixon sequence. It is reasonable to believe that the epicardial fat segmentation for the single-phase Dixon could be overestimated due to the inclusion of the adjacent paracardial fat if the border between these two adipose tissue depots is diffuse or blurred in that particular cardiac phase. Conversely, for the cine-Dixon, the border may be more precisely inferred by interpolating from alternative time frames where it may be more clearly visible, as shown in Figs. 2 and 3 and supplementary video.

Body mass index is a common anthropometric index of overweight and obesity. Interestingly, epicardial fat volume index to BSA showed a significant relation to BMI using the proposed cine Dixon technique but not using the single-phase technique. However, the relation was only modest implying that BMI is a rather unspecific metric of ectopic adipose tissue [6]. In previous studies using CMR Dixon imaging of non-cardiac adipose tissue depots, not only the fat volume but also the fat concentration has been assessed [30]. This information has not yet been obtained for cardiac adipose tissue but would likely provide additive value in terms risk assessment of cardiovascular disease.

Compared to previous work using single-phase Dixon for fat quantification by Homsi et al. [14], we have obtained a similar mean measurement error (bias) for the inter-observer variability comparison (0.42 ml per slice × 10 slices per patient = 4.2 ml per patient in this study vs. 4.5 ml in Homsi et al.) but with a higher standard deviation (1.53 ml per slice in this study, 15.3 ml per patient vs. 4.1 ml in Homsi et al.). This may be due to different acquisition protocols, where the images in this study were acquired after contrast injection while in Homsi et al. the Dixon images were acquired without contrast.

The implemented fat quantification methods rely on the fat fraction variable to classify voxels as fat [14]. The advantage of using the fat fraction for quantification is that flow-related artifacts which may otherwise be classified as fat are suppressed and simple threshold-based segmentation techniques may be readily applied. Compared to previous work which use a subject-specific threshold obtained by segmenting areas of pure fat and myocardium [14], we used a fixed threshold for all datasets. This minimizes variability in quantification due to differences in segmentation of the fat and myocardium region-of-interests which may confound the variability of segmentation of the epicardial fat. Due to the low steady-state signal of water signal for the spoiled gradient recalled-echo sequence used here, a T1-shortening contrast agent was necessary to facilitate the fat quantification for the cine Dixon technique. However, the fat signal is unaffected by the contrast agent and the use of a contrast agent is therefore not a strict requirement, its primary purpose is to support the classification problem. Classification methods that only utilize the fat signal can be developed which relaxes this requirement.

A potential bias with regard to the contrast agent administration could be caused by the non-randomization of scan order (cine Dixon was always performed first to maximise contrast agent effect, while the single phase scan was performed second as it does not benefit from contrast agents to the same extent). However, the contrast agent (gadobutrol) has a relatively long washout period and a half-life of approximately 2 h [31]. The 5 to 7-min difference in time after contrast agent injection between cine and single-phase Dixon therefore is unlikely to significantly affect the outcomes.

Accurate assessment of epicardial adipose tissue using the proposed cine 3D Dixon technique could be a valuable complement to cardiac fibrosis assessment using late gadolinium enhancement, as there is a link between epicardial adipose tissue, inflammation, and cardiac fibrosis [32]. Furthermore, automatic segmentation of the cine 3D Dixon based epicardial adipose tissue volume would improve the clinical applicability, and this is ongoing work.

A limitation of the cine Dixon technique compared to single-phase Dixon is the longer scan time which is due to the higher temporal resolution of cine Dixon. In this study we used a temporal resolution of 65 ms for the cine Dixon scan, while the single-phase resolution was approximately 100 ms, leading to a proportional increase in scan time. The lower temporal resolution of the single-phase Dixon is unlikely to significantly reduce image quality as it was adapted to coincide with the patient-specific rest period. Maintaining a high temporal resolution for cine Dixon is important to mitigate cardiac motion blurring and capitalize on the captured cardiac motion between frames which facilitates delineation of the epicardial fat border. However, by employing compressed sensing with high image acceleration factors a nominal scan time of 2 min and 45 s was achieved, which was considered clinically acceptable. For comparison, the nominal scan time for single-phase Dixon was 1 min and 50 s, which is a 50% reduction compared to cine Dixon.

Another limitation of this study is the small number of patients that were scanned. Although the aim of this proof-of-concept study was primarily to demonstrate that cine Dixon may be a more robust approach than single-phase Dixon, further studies are required to evaluate the clinical value of this technique in a larger patient cohort and fat values obtained with cine Dixon to techniques which are used more routinely to quantify epicardial fat such as computed tomography [33].

## Conclusion

A new time-resolved cine Dixon technique can be used to quantify epicardial fat with a lower intra- and inter-observer variability compared to the standard single-phase Dixon technique. Despite a small reduction in image quality of the water images compared to single-phase Dixon, the information provided by the time-resolved acquisition appears to support the delineation of the border of the epicardial adipose tissue depot.

## Supplementary information

**Additional file 1: Video S1.** Time-resolved cine Dixon scan of patient 1 from Fig. 2.

**Additional file 2: Video S2.** Time-resolved cine Dixon scan of patient 2 from Fig. 2.

**Additional file 3: Video S3.** Time-resolved cine Dixon scan of the patient from Fig. 3.

## Data Availability

The anonymized data that support the findings of this study are available from the corresponding author on reasonable request.

## Abbreviations

CMR: cardiovascular magnetic resonance
3D: three-dimensional
TR: repetition time
TE_1_: first echo time
TE_2_: second echo time
W: water image
F: fat image
FF: fat fraction image
ICC: intraclass correlation coefficient
BMI: body mass index
BSA: body surface area
